# Exploring the mediating role of social support and learning engagement in the relationship between physical activity and academic achievement in secondary school students

**DOI:** 10.3389/fpsyg.2025.1387475

**Published:** 2025-04-09

**Authors:** Jun Xiang, Kelei Guo, Jia Gao, Yun Gao, ShiLei Liu

**Affiliations:** ^1^School of Physical Education and Health, Zhaoqing University, Zhaoqing, China; ^2^Department of Physical Education, Shanghai Jiao Tong University, Shanghai, China; ^3^School of Physical Education and Health, Wenzhou University, Wenzhou, China

**Keywords:** physical activity, academic achievement, social support, learning engagement, mediation, secondary school students

## Abstract

**Background:**

The academic achievement of secondary school students has consistently been a focal topic of interest among researchers. However, the relationship between physical activity and academic achievement, along with its underlying mechanisms, remains unclear. Therefore, the purpose of this study was to investigate the relationship between physical activity and academic achievement of secondary school students, and to verify the mediating role of social support and learning engagement between them.

**Methods:**

Based on the purpose, a survey was conducted involving 3,230 secondary school students (*M* age = 13.21, *SD* age = 0.54) in Guangdong Province, utilizing the Physical Activity Level Scale, Academic Achievement Scale, Perceived Social Support Scale, and Learning Engagement Scale. Data were statistically analysed using descriptive statistics, correlation analysis, regression analysis and mediation analysis by using SPSS to examine the relationship between physical activity and academic achievement, as well as the mediating roles of social support and learning engagement.

**Results:**

Independent Sample *t*-test were used to test gender differences, which were observed only in physical activity, with boys exhibiting significantly higher scores than girls (Boys: 36.41 ± 19.17; Girls: 34.21 ± 19.78; *p* = 0.008). ANOVA were used to test age differences, which were observed in physical activity (*F* = 3.426, *p* = 0.001) and learning engagement (*F* = 3.054, *p* = 0.012), with physical activity declining among middle school students as age increased, while learning engagement showed a continuous rise across all age stages. Regression analysis showed that the direct path from physical activity to academic achievement was significant (β = −0.025, *p* < 0.01). Physical activity positively predicted social support (β = 0.085, *p* < 0.01) and learning engagement (β = 0.082, *p* < 0.01). Social support significantly predicted learning engagement (β = 0.096, *p* < 0.01) and academic achievement (β = −0.038, *p* < 0.01). Social support and learning engagement play significant mediating roles in the relationship between physical activity and academic achievement, accounting for 90.25% of the total effect. The mediating effect consists of three pathways: (1) physical activity → social support → academic achievement (mediating effect is 0.017), (2) physical activity → learning engagement → academic achievement (mediating effect is 0.032), and (3) physical activity → social support → learning engagement → academic achievement (mediating effect is 0.062).

**Conclusion:**

Physical activity not only directly predicts academic achievement in middle school students, but also directly through the separate mediating roles of social support and learning engagement, and indirectly through the chained mediating roles of social support and learning engagement. These findings underscore the significant influence of physical activity on academic achievement, offering valuable insights for educators in developing and implementing strategies that foster students’ academic development.

## Introduction

In July 2021, the General Office of the Central Committee of the Communist Party of China issued the “Opinions on Further Reducing the Burden of Homework and Extracurricular Training for Students in Compulsory Education.” This document advocated for the effective use of students’ leisure time, encouraging schools and parents to guide students in engaging in manageable household chores and appropriate physical exercise after school (Zhang et al., 2023). With increasing societal competition and ongoing educational reforms, stakeholders—including schools, parents, and students—continue to prioritize academic performance due to the influence of traditional examination-oriented education. This often results in a substantial investment of time and energy in cultural subjects, leading to a neglect of the importance of physical activity (Zhao et al., 2024). Over time, this has manifested in the frequent reallocation of physical education class hours to cultural subjects, resulting in students lacking sufficient opportunities for physical activity (Wang et al., 2023).

However, studies have shown that physical activity not only imparts motor skills and enhances students’ physical fitness but also contributes to improved cognitive processing and executive functions, thereby enhancing students’ mathematical performance (Ericsson and Karlsson, 2014). Longitudinal studies have further demonstrated that students who maintain good or improved levels of aerobic endurance tend to achieve higher scores on various academic assessments (Sardinha et al., 2016; Andersen et al., 2016). Moreover, engaging in physical exercise promotes students’ physical health and facilitates the release of various neurotransmitters in the brain, which not only induces feelings of pleasure and enhances cognitive flexibility but also increases arousal levels and cognitive resources. This, in turn, fosters the development of a positive self-concept among students, thereby improving their psychological well-being and academic success (Jiang et al., 2016).

Given that middle school students face increasing academic pressures, physical activity may serve as an effective intervention to promote academic achievement (Gao et al., 2024). Few empirical studies have investigated the mechanisms underlying the relationship between physical activity and academic achievement. Therefore, this study aims to investigate the mechanisms through which physical activity influences academic achievement among secondary school students, while also analyzing the mediating roles of social support and learning engagement between the two variables. This research seeks to provide both theoretical and empirical evidence to enhance academic performance and foster academic engagement among secondary school students.

## Literature view and research hypothesis

### Physical activity and academic achievement

Recent studies highlight physical activity as a crucial factor influencing adolescents’ overall well-being (Sun et al., 2021), demonstrating positive effects on both their physical and mental health (Pascoe et al., 2020). Specifically, higher levels of physical activity are linked to improved academic achievement, with active students often exhibiting greater motivation, self-discipline, and focus in their studies (Estrada-Tenorio et al., 2020). Engaging in moderate-intensity activities, such as jogging or swimming, has been shown to significantly enhance math and language scores (Singh et al., 2012). Additionally, physical activity reduces stress and anxiety, enhancing students’ emotional states and focus during study sessions (Erdoğan Yüce and Muz, 2020). Therefore, the current study proposed Hypothesis 1: physical activity is positively associated with academic achievement in secondary school students.

### The mediating role of social support

One of the mediating mechanisms in this study is the role of social support. Social support refers to support and encouragement from social relationships such as family members, teachers, and peers (Gottlieb and Bergen, 2010). First, social support plays an important role in an individual’s academic achievement (Li et al., 2018). Social support from family, peers, and teachers can enhance students’ motivation, self-confidence, and satisfaction with learning, thereby improving their academic performance (Toor, 2018). Fredricks and Eccles (2006) found that social support can increase students’ motivation, which in turn promotes academic achievement. Second, engaging in physical activities, whether in team sports, group fitness classes, or recreational activities, brings people together with a common interest. These shared experiences facilitate interactions, which can lead to the formation of friendships and social networks (Fan et al., 2011). Therefore, the current study proposed Hypothesis 2: social support has a mediating role between physical activity and academic achievement of secondary school students.

### Mediating role of learning engagement

The second mechanism of mediation in this study is the mediating role of learning engagement. Learning engagement refers to students’ attention, commitment, and motivation to the learning task (Jang, 2008). A growing body of research indicates that physical activity exerts a positive influence on students’ attitudes, attention, and motivation toward learning, which subsequently enhances their engagement and academic performance. For instance, de Bruijn et al. (2023) demonstrated that physical activity fosters students’ attention and commitment to learning tasks, which in turn contributes to improved academic achievement. Similarly, Hagger et al. (2002) and Wiltshire et al. (2019) reported that students who engage in physical activity are more motivated and actively involved in their studies, yielding positive academic outcomes. Therefore, the current study proposed Hypothesis 3: learning engagement mediates between physical activity and academic achievement of secondary school students.

### Chain mediating role of social support and learning engagement

Social support and learning engagement may have a chain mediating role between physical activity and academic achievement. Social support can increase students’ self-esteem and self-confidence, which in turn increases their commitment and effort to academic tasks, social support can affect academic achievement by influencing learning engagement (Pfeifer et al., 2023). Therefore, the current study proposed Hypothesis 4: social support and learning engagement serve as chain mediating roles between physical activity and academic achievement of secondary school students.

**Figure 1:**
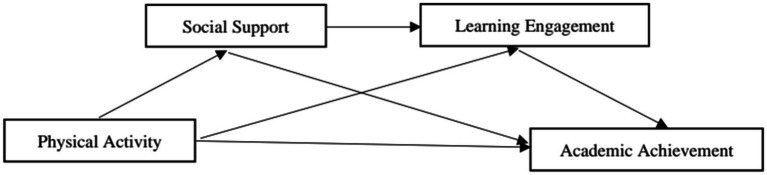
Research frame diagram.

In summary, scholars have pointed out the positive association between physical activity and academic achievement and emphasized the mediating and chain mediating roles of social support and learning engagement in this relationship, and the above findings provide a theoretical basis for the hypotheses of this study. Therefore, the research frame diagram were established (Figure 1): (1) to test the predictive role of physical activity on secondary school students’ academic achievement; (2) to examine the mediating role of social support between physical activity and secondary school students’ academic achievement; (3) to examine the mediating role of learning engagement between physical activity and secondary school students’ academic achievement; (4) to test the chain-mediating roles of social support and learning engagement in the relationship between physical activity and secondary school students’ academic achievement. Chain mediating role.

## Method

### Participant

To enhance the representativeness of the sample, factors such as economic status were taken into consideration. Based on the current administrative division of Guangdong Province, two secondary schools (42 in total) were randomly selected from each of the 21 prefecture-level cities in Guangdong Province, including Guangzhou, Shenzhen, Zhuhai, Shantou, and Foshan, as the sample sampling schools for this study. Two classes (Approximately 40–42 students are selected from each class) were randomly selected from each school (a total of 84 classes) and 3,528 questionnaires were distributed. The subjects were tested in the physical education classroom and the primary testers were professionally trained physical education teachers and psychology students. The tests were approved by the school director, classroom teachers and subjects, and all questionnaires were completed within 10 min. A group-based administration method was employed, with standardized instructions provided to guide participants in completing the questionnaires. After the questionnaires were recovered, they were excluded according to the following criteria: (1) missing data (e.g., participants did not complete the majority of the items); (2) inconsistent responses (e.g., participants provide answers that are logically contradictory); (3) absence of participation in physical exercise or limitations in physical activity; and (4) refusal by participants or their legal guardians to consent to participation. After collation 3,230 valid questionnaires were recovered for this study with a recovery rate of 92%. The age range of the participants was 12 to 17 years (*M* age = 13.21, *SD* age = 0.54), of which 1,578 were boys and 1712 were girls. The sample size was computed *a priori* by G∗Power 3.1 (Faul et al., 2007), using the power of (1-β) = 0.95, medium effect size|𝜌| = 0.3 of the expected variable-correlations and a two-tail test. The recommended sample size by G∗Power calculation was 134 and we obtain reliable statistical calculations. In addition, the study was supported and approved by the Institutional Review Board of Zhaoqing University. All secondary school and the student’s families signed an informed consent form. The informed consent form described the purpose and process of the study, the methodology used, and also included information on the assurance of confidentiality, the principle of voluntary participation, and the contact information of the researchers. The variables such as gender and class of the subjects were also controlled.

### Research methods

#### Psychometric methods

##### Physical activity rating scale

The Physical Activity Rating Scale (PARS-3) compiled by Liang (1994) was chosen, which is widely used within the field of physical activity, and it is mainly used to assess an individual’s level of physical activity. The PARS-3 is a self-reported scale and consists of three dimensions: intensity of activity (e.g., How would you describe the intensity of your physical exercise?), duration of activity (e.g., How much time do you spend on exercise each time?), and number of activities (e.g., How many times do you participate in this exercise activity?). Each item is divided into five levels and scored 1–5 points. Physical activity exercise = intensity × time × frequency, and the amount of physical activity is converted to a maximum of 100 points and a minimum of 0. The criteria for the development of the Physical Activity Scale are as follows: low activity ≤ 19 points; slightly low activity ≤ 20–39 points; moderate activity ≤ 40–59 points; slightly high activity ≤ 60–79 points; high activity ≤ 80–100 points. Previous study proved that the scale has good reliability and validity in the middle school student population (Yan et al., 2020). In this study, the Cronbach’s α coefficient of the scale is 0.51, which indicates that the scale questionnaire has acceptable reliability and validity, although it is lower than the value reported in Yan’s study (α = 0.76; Yan et al., 2020).

##### Academic achievement scale

The Academic Achievement Questionnaire developed by Wen et al. (2010) was used to ask students to evaluate their academic achievement in 3 subjects, namely, language, mathematics and English. The response options incorporated a 5-point Likert scale, which ranged from 1 (very bad) to 5 (very good), and the average score of the three subjects was finally calculated. In addition, considering the combined calculation of the test scores of the two subjects, this study did a score conversion of the raw test scores during data processing (mean 50, standard deviation 10), and the higher the score, the stronger the academic achievement of the subjects (Zhu, 2014). Previous study proved that the scale has good reliability and validity in the secondary school student population (Lin, 2021). In this study, the Cronbach’s α coefficient of the scale is 0.88, similar to Lin’s study (α = 0.72), which indicates that the scale questionnaire has good reliability and validity.

##### Social support scale

The Perceived Social Support Scale was originally developed by Zimet et al., 1988 and later translated and revised by Jiang (2009). The scale consists of 12 questions in 3 dimensions (family support, friend support, and other support). The response options incorporated a 5-point Likert scale, which ranged from 1 (Strongly Disagree) to 5 (Strongly Agree). Higher total scores indicating higher levels of social support. Research has proved that the scale has good reliability and validity in the secondary school student population (Xu, 2023). In this study, the Cronbach’s alpha coefficient of the scale is 0.879, similar to Xu’s study (α = 0.89), which indicates that the scale questionnaire in this study has good reliability and validity.

##### Learning engagement scale

The Learning Engagement Scale developed by Schaufeli et al. (2002) revised by Fang et al. (2008) was used. The revised scale consists of three dimensions: vigor (6 items), dedication (5 items), and concentration (6 items), with a total of 17 items. The response options incorporated a 7-point Likert scale, which ranged from 1 (never) to 5 (always). Higher total scores indicating higher levels of learning engagement. Research has proved that the scale has good reliability and validity (Gong, 2022). In this study, the Cronbach’s alpha coefficient of the scale is 0.92, similar to Gong’s study (α = 0.97), which indicates that the scale questionnaire in this study has good reliability and validity.

##### Data collecting procedure

First, 1 week prior to the formal signing of the informed consent forms by the middle school students, the research team distributed guardian versions of the informed consent forms to 3,528 students across 84 selected classes. The consent forms informed guardians about the purpose, significance, and content of the study (students were instructed to explain the contents of the consent forms to their parents and to inform them that they needed to sign). Additionally, students from the selected classes were required to bring the guardian versions of the consent forms back to school on the day of the formal testing and submit them to the testing staff. Second, on the day of the formal testing, middle school students were given ample time and opportunity to review the details of the test before signing the informed consent form. Researchers thoroughly answered any questions related to the study posed by the participants. Due to factors such as the large number of students selected from classes, the need for guardians to sign the consent forms before testing, and the varying schedules for physical education classes, the testing process ultimately collected participants’ basic information (age, gender, etc.), as well as their evaluations of physical activity, academic achievement, social support, and learning engagement. The entire process took a month to complete.

##### Mathematical and statistical method

After the questionnaire of this study was recovered and screened again to exclude invalid data, the data of this study were statistically analyzed using SPSS21.0 and SPSS macro program prepared by Hayes. The SPSS PROCESS macro is a widely used tool for mediation and moderation analysis, allowing for a clear examination of complex relationships among variables (Hayes and Aut, 2018). Previous studies have used this method to analyze mediating effects (e.g., Liu et al., 2024). First, descriptive statistics with test of difference were performed on the test data of demographic information, physical activity, academic achievement, social support, and commitment to learning using SPSS 21.0 software. Second, common method bias was tested using public latent factors in SPSS 21.0 software. Third, Pearson’s bivariate relationship between physical activity, academic achievement, social support and learning engagement among secondary school students was tested using SPSS 21.0. Fourth, PROCESS model 6 and Bootstrap (5,000 times) sampling technique were utilized to test the mediating roles of social support and learning engagement, and the chain mediating role between physical activity and academic achievement after control gender and age. In this study, *p* < 0.05 was set as statistical result and significant.

## Research results

### Common method bias test

Data collection using questionnaires may be at risk of common method bias. Therefore, before analyzing the data, Harman’s one-factor test was used for statistical control, i.e., the items of all variables were subjected to unrotated principal component factor analysis (Zhou and Long, 2004). The results showed that there were six factors with eigenvalues greater than 1 extracted from the results of the unrotated exploratory factor analysis, and the variance of variance explained by the first factor was 29.80%, which was less than the critical value of 40% (Yan et al., 2020). It means that there is no factor that can explain most of the variance in this study, so the data in this study does not have serious common method bias and meets the conditions for further chained mediation effect test.

### Descriptive statistics of physical activity, academic achievement, social support and learning engagement

The statistical results in Table 1 show that physical activity, academic achievement, social support, and learning engagement are not statistically significant when analyzed by gender differences (*p* > 0.05); physical activity, academic achievement, and learning engagement are statistically significant when analyzed by age differences (*p* < 0.05), and social support is not statistically significant when analyzed by age differences (*p* > 0.05). Boys scored slightly higher than girls in the physical activity and social support tests, while they scored slightly lower than girls in the academic achievement and learning engagement tests. In terms of physical activity, the amount of exercise among middle school students tends to decline with age. In contrast, regarding learning engagement, there is a continuous increase in engagement across all age stages as age increases. In terms of academic achievement and social support, there are no significant changes in academic performance across different age stages (see Table 2).

**Table 1:** Gender differences in physical activity, academic achievement, social support, and learning engagement.

Variable	Gender	Number (%)	*M*	SD	*t*	*p*
Physical activity	Male	1,518 (47%)	36.41	19.17	2.523	0.008
	Female	1,712 (53%)	34.21	19.78		
Social support	Male	1,518 (47%)	52.13	11.32	1.378	0.375
	Female	1,712 (53%)	51.93	11.36		
Learning engagement	Male	1,518 (47%)	67.39	16.71	0.969	0.613
	Female	1,712 (53%)	66.19	16.12		
Academic achievement	Male	1,518 (47%)	53.67	1.00	0.582	0.841
	Female	1,712 (53%)	53.67	0.97		

**Table 2:** Age difference of physical activity, academic achievement, social support and learning engagement test results.

Variable	Age	Number (%)	*M*	SD	*F*	*p*
Physical activity	12	485 (15.02%)	42.28	18.59	3.426	0.001
	13	1,130 (34.98%)	39.98	18.86		
	14	452 (13.99%)	38.74	16.51		
	15	388 (12.01%)	39.55	17.24		
	16	419 (12.97%)	22.51	4.63		
	17	356 (11.02%)	20.33	4.78		
Social support	12	485 (15.02%)	49.96	11.67	2.109	0.061
	13	1,130 (34.98%)	52.14	11.34		
	14	452 (13.99%)	51.89	11.35		
	15	388 (12.01%)	54.23	6.11		
	16	419 (12.97%)	54.61	4.35		
	17	356 (11.02%)	42.67	15.61		
Learning engagement	12	485 (15.02%)	62.79	14.39	3.054	0.012
	13	1,130 (34.98%)	66.77	16.35		
	14	452 (13.99%)	67.82	16.62		
	15	388 (12.01%)	68.44	21.82		
	16	419 (12.97%)	70.39	14.86		
	17	356 (11.02%)	73.78	17.34		
Academic achievement	12	485 (15.02%)	53.96	0.76	2.157	0.057
	13	1,130 (34.98%)	53.65	0.98		
	14	452 (13.99%)	53.68	1.12		
	15	388 (12.01%)	53.29	1.33		
	16	419 (12.97%)	53.89	0.84		
	17	356 (11.02%)	53.89	1.32		

### Correlation analysis of physical activity, academic achievement, social support and learning engagement

As can be seen from Table 3, physical activity showed significant positive correlations with academic achievement, social support, and learning engagement. Additionally, academic achievement was positively correlated with both social support and learning engagement, and a significant correlation was found between social support and learning engagement.

**Table 3:** Correlation analysis and statistics of physical activity, academic achievement, social support and learning engagement.

Variable	*M* ± *SD*	Physical activity	Academic achievement	Social support	Learning engagement
Physical activity	36.04 ± 19.49	1			
Academic achievement	53.67 ± 0.99	0.37^**^	1		
Social support	52.03 ± 11.34	0.58^**^	0.55^**^	1	
Learning engagement	67.13 ± 16.40	0.43^**^	0.89^**^	0.66^**^	1

*N* = 3,230. **p < 0.01.

### Testing the mediating effects of social support and learning engagement

Physical activity was taken as the independent variable, social support and learning involvement as the mediating variable, and academic achievement as the dependent variable. The SPSS macro model 6 was adopted (Hayes and Aut, 2018), and non-parametric percentile Bootstrap test with deviation correction was selected (repeated sampling 5,000 times). A 95% confidence interval was calculated to analyze the effects of the chain mediation model (Wen et al., 2022). After control gender and age, the results showed that physical activity positively associated with academic achievement, β = 0.021, *p* < 0.01, supporting Hypothesis 1. Physical activity was positively associated with social support, β = 0.013, *p* < 0.01. Social support was positively associated with academic achievement, β = 0.043, *p* < 0.01, supporting Hypothesis 2. Physical activity was positively associated with learning engagement, β = 0.52, *p* < 0.01. Learning engagement was positively associated with academic achievement, β = 0.095, *p* < 0.01, supporting Hypothesis 3. Social support was positively associated with learning engagement β = 0.073, *p* < 0.01, supporting Hypothesis 4 (see Table 4).

**Table 4:** Regression analysis of the chain mediation model between physical activity and academic achievement.

Variable	Social support		Learning engagement		Academic achievement	
	β	*t*	β	*t*	β	*t*
Gender	0.032	6.26^**^	0.047	7.81^**^	0.004	0.914
Age	0.021	6.221^**^	0.043	7.626^**^	0.051	8.013^**^
Physical activity	0.033	6.331^**^	0.072	11.013^**^	0.059	8.422^**^
Social support			0.083	12.086^**^	0.063	9.573^**^
Learning engagement					0.095	13.192^**^
*R* ^2^	1.921		1.473		1.814	
*F*	123.242		143.941		253.883	

^*^P < 0.05, ^**^P < 0.01.

**Figure 2:**
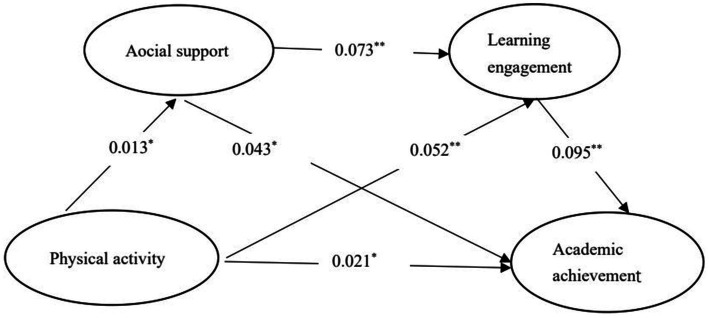
The chain mediation model of social support and learning engagement between physical activity and academic achievement.

The results of the mediation effect test are shown in Table 5, Bootstrap 95% confidence interval of the indirect effects of physical activity on academic achievement does not include 0. These findings further support Hypotheses 2 to 4 (Figure 2).

**Table 5:** Tests the mediating effects of social support and learning engagement on physical activity and academic achievement.

Effect type	Effect size	Boot SE	Bootstrap95% CI		Effect ratio
			Floor	Upper limit	
Total effect	0.123	0.089	0.029	0.052	100%
Direct effect	0.012	0.039	0.015	0.036	9.76%
Physical activity - Social support - Academic achievement	0.017	0.090	0.035	0.070	13.82%
Physical activity - Learning engagement - Academic achievement	0.032	0.053	0.015	0.075	26.02%
Physical activity - Social support - Learning engagement - Academic achievement	0.062	0.077	0.012	0.021	50.41%
Total indirect effect	0.111	0.078	0.011	0.023	90.24%

## Discussion

As predicted, the current study found that (1) physical activity positively associated with academic achievement; (2) Social support and learning engagement mediated the relationship between physical activity and academic achievement.

### Physical activity and academic achievement

The results of this study show that there is a positive relationship between physical activity and academic achievement, which is consistent with numerous earlier studies (Sallis et al., 2012; Coe et al., 2006). First, engaging in physical activities enhances cognitive function and attention, leading to improved learning capabilities (Sallis et al., 2012). Moreover, physical activity contributes to the enhancement of working memory and the speed of information processing, which are positively correlated with academic achievement (Jiang et al., 2016). Secondly, physical activity promotes physical health and psychological well-being, enhancing students’ motivation and learning effectiveness. Regular participation in physical activities helps students maintain good health, increases fitness levels, and boosts endurance, resulting in greater energy and concentration in their studies (Coe et al., 2006).

Furthermore, the results of this study indicate that there are gender differences in physical activity among middle school students. Boys scored slightly higher than girls in physical activity. A possible explanation is that boys are often encouraged to participate in more vigorous physical activities and sports, resulting in higher activity levels (Ridgers et al., 2006). Culturally, there may be stereotypes that associate physicality and competitiveness with masculinity, motivating boys to participate more in sports and physical activities (Telford et al., 2016).

### Mediating role of social support

The results of this study show that social support plays a mediating role between physical activity and academic achievement, which indicated that physical activity is positively associated with social support, thereby increasing students’ academic achievement. This is consistent with the findings of Fredricks and Eccles (2006) and Hagger et al. (2002). Social support can enhance students’ motivation and opportunities to engage in physical activity by providing encouragement and assistance, thereby facilitating improvements in academic achievement. Researchers have found that the perception of family support can enhance students’ levels of physical activity, and increased physical activity is positively correlated with students’ academic performance. Therefore, it is believed that family support can improve academic achievement by promoting students’ physical activity (Barbosa et al., 2020). Scholars have also discovered a significant positive relationship between teacher support and students’ physical activity levels and academic achievement, as teacher support can stimulate students’ interest in engaging in physical activities and provide relevant opportunities and guidance (Vella et al., 2015). Additionally, research has shown that perceived peer support is significantly positively associated with students’ physical activity levels and academic achievement. Social support comes not only from family and school environments but also from social networks and communities. Friends and peers in students’ social networks can influence their physical activity and academic success (Anderson et al., 2006). Therefore, in the process of improving students’ academic achievement, it is essential to emphasize social support, providing students with ample encouragement and support.

### Mediating role of learning engagement

The results of this study show that learning engagement plays a mediating role between physical activity and academic achievement, which means that learning engagement can indirectly contribute to students’ academic achievement by influencing their participation in physical activity. These findings align with previous studies examining mediating factors in academic performance (Diotaiuti et al., 2021), which emphasized the role of emotional balance and procrastination in shaping students’ outcomes. Studies show that students who engage in physical activities exhibit higher levels of learning motivation, learning goals, and the use of learning strategies. Physical activity can enhance students’ autonomy and self-regulation skills, thereby promoting their learning engagement (Nurmi et al., 2016). Additionally, physical activity can facilitate students’ flow experiences, characterized by concentration and immersion in the context (Beauchamp and Morton, 2011). Furthermore, research has found that learning engagement significantly predicts academic achievement (Fredricks et al., 2004). Thus, learning engagement is considered a crucial bridge between physical activity and academic success. By promoting students’ learning engagement, physical activities can positively impact their academic development.

### Chain mediating role of social support and learning engagement

The results of this study indicate that social support and learning engagement play an important chain-mediated role between physical activity and academic achievement. This is consistent with previous research findings (Hillman et al., 2008). Social support can provide learning resources and guidance, which in turn affects students’ levels of learning engagement. Support from family, peers, and teachers not only offers learning resources and information but also provides guidance and assistance (Leo et al., 2022). Social support can create opportunities for resource and information sharing, enhancing students’ motivation and academic achievement (Wu et al., 2023; Meece et al., 2006). When students encounter learning difficulties or setbacks, receiving support and encouragement from others helps them adjust their learning attitudes and boost their motivation, leading to more active participation in learning activities.

### Practical implications

The results of this study highlight the importance of promoting physical activity as a key element in enhancing academic achievement among secondary school students. Given the chain-mediating role of social support and learning engagement, educators and policymakers should consider adopting a more holistic approach to student development. Schools should integrate structured physical activity programs into the daily schedule, such as offering more diverse physical education classes and encouraging extracurricular sports activities. In parallel, schools should work to build supportive environments by strengthening peer relationships, fostering positive teacher-student connections, and creating systems that promote social support among students. This could include mentorship programs, team-based projects, or peer support networks.

Furthermore, by recognizing the positive impact of learning engagement, educators can develop strategies to make academic tasks more interactive and engaging. Policymakers can also allocate resources toward training teachers to create active learning environments and provide more personalized feedback, which could further enhance students’ involvement in their learning process.

### Limitation and suggestions for future study

This study provides a theoretical contribution to the study of academic achievement of secondary school students, the role of physical activity in intervening in the academic achievement of secondary school students as well as in increasing social support and facilitating learning engagement. However, it also has the following shortcomings:

1. Sample limitation: The sample is limited to one region (Guangdong), so the findings may not be generalizable to other areas. Future research could consider expanding the sample size to include students from more regions and diverse backgrounds to enhance the external validity of the findings.
2. Self-reported data: The measurements rely on questionnaires, which may introduce biases due to students’ self-perception. To improve the objectivity and reliability of the findings, future research could incorporate objective measurement indicators, such as physical activity monitors and academic performance records, to obtain more accurate data.
3. Possible interfering variables: Factors such as socioeconomic status, study hours, or quality of instruction were not controlled. Future research could control for these potential confounding factors to obtain more accurate results.
4. Long-term effects: This study primarily focused on the relationship between physical activity and middle school students’ academic achievement but did not consider long-term effects. Future research could conduct longitudinal studies to understand the long-term impact of physical activity on academic achievement and determine whether the mediating roles of social support and learning engagement remain stable at different time points.

## Conclusion

This study explores the relationship between physical activity and academic achievement among middle school students, verifying the mediating roles of social support and learning engagement between the relationship between these two variables. First, there are gender and age differences in physical activity among middle school students. Second, here is a significant positive relationship between physical activity and academic achievement in middle school students, indicating that physical activity has a positive impact on their academic achievement. Third, physical activity not only directly predicts academic achievement but also indirectly predicts it through the chain mediating effects of social support and learning engagement. Based on these findings, schools and educational institutions should implement measures to promote student participation in physical activities, enhance social support, and foster a positive learning environment to improve academic development.

## Data Availability

The raw data supporting the conclusions of this article will be made available by the authors, without undue reservation.
